# A Free Radical Scavenger Ameliorates Teratogenic Activity of a DNA Hypomethylating Hematological Therapeutic

**DOI:** 10.1089/scd.2018.0194

**Published:** 2019-06-03

**Authors:** Nikola Sobočan, Ana Katušić Bojanac, Nino Sinčić, Marta Himelreich-Perić, Jure Krasić, Željka Majić, Gordana Jurić-Lekić, Ljiljana Šerman, Maja Vlahović, Davor Ježek, Floriana Bulić-Jakuš

**Affiliations:** ^1^ Department of Gastroenterology, School of Medicine, University Hospital Merkur, University of Zagreb, Zagreb, Croatia.; ^2^ Center of Excellence in Reproductive and Regenerative Medicine, School of Medicine, Zagreb, Croatia.; ^3^ Department of Medical Biology, School of Medicine, University of Zagreb, Zagreb, Croatia.; ^4^ Department of Histology and Embryology, School of Medicine, University of Zagreb, Zagreb, Croatia.

**Keywords:** 5-azacytidine, *N*-tert–butyl-α-phenylnitron, embryo, teratogenesis, ROS/RNS

## Abstract

The spin-trap free radical scavenger *N*-tert-butyl-α-phenylnitron (PBN) ameliorated effects of several teratogens involving reactive oxygen species (ROS). We investigated for the first time whether PBN could ameliorate teratogenesis induced by a DNA hypomethylating hematological therapeutic 5-azacytidine (5azaC). At days 12 and 13 of gestation, Fisher rat dams were pretreated by an i.v. injection of PBN (40 mg/kg) and 1 h later by an i.p. injection of 5azaC (5mg/kg). Development was analyzed at gestation day 15 in embryos and day 20 in fetuses. PBN alone did not significantly affect development. PBN pretreatment restored survival of 5azaC-treated dams' embryos to the control level, restored weight of embryos and partially of fetuses, and partially restored crown-rump lengths. PBN pretreatment converted limb adactyly to less severe oligodactyly. PBN pretreatment restored global DNA methylation level in the limb buds to the control level. Cell proliferation in limb buds of all 5azaC-treated dams remained significantly lower than in controls. In the embryonic liver, PBN pretreatment normalized proliferation diminished significantly by 5azaC; whereas in embryonic vertebral cartilage, proliferation of all 5azaC-treated dams was significantly higher than in PBN-treated dams or controls. Apoptotic indices significantly enhanced by 5azaC in liver and cartilage were not influenced by PBN pretreatment. However, PBN significantly diminished ROS or reactive nitrogen species markers nitrotyrosine and 8-hydroxy-2′deoxyguanosine elevated by 5azaC in embryonic tissues, and, therefore, activity of this DNA hypomethylating agent was associated to the activation of free radicals. That pretreatment with PBN enhanced proliferation in the liver and not in immature tissue is interesting for the treatment of 5azaC-induced hepatotoxicity and liver regeneration.

## Introduction

We have previously shown that a DNA hypomethylating agent 5-azacytidine (5azaC), administered during the rat gestation in vivo, significantly impaired fetal growth, induced specific malformations, and disrupted placental development and its glycoprotein composition [1,2]. Although 5azaC negatively affects placenta, it may directly affect development of the embryo itself without any extraembryonic tissues in vitro [3,4]. We have also previously shown that the acetylsalicylic acid (ASA) ameliorated teratogenic impact on the offspring of 5azaC-treated dams in vivo [5,6]. ASA is a free radical scavenger and inhibitor of the peroxidase-cyclooxygenase component of the prostaglandin H synthetase. It has been effective in the inhibition of phenytoin-initiated oxidative damage to protein and lipids in embryonic tissues [7].

Another free radical scavenger the spin-trap, *N*-tert-butyl-α-phenylnitron (PBN), exerted similar activity as ASA [8], being protective against several teratogens that involve reactive oxygen species (ROS) such as phenytoin, benzo[a]pyrene, thalidomide, ethanol, and methanol [9,10]. PBN itself has been used for the detection and identification of many other short-lived free radicals that react covalently with PBN and form more stable adducts to be recognized by electron paramagnetic resonance spectroscopy [8]. The understanding of the full activity of PBNs is difficult because it also reacts with methyl, methanol sulfite, azidyl, cyanatyl, and cyanyl radicals and chlorine atoms [11], carotenoid radicals, retinil radicals etc. [12].

As the best known role of nitrones is to scavenge anion superoxide and hydroxyl radicals, they are sometimes classified as antioxidant therapeutics proposed for a wide variety of medicinal indications and have been used for therapy of various conditions such as neurodegeneration, cardiovascular disease, stroke, and cancer [13,14], in antipollution cosmetics, noise-induced neural degeneration, elimination of nociceptive behavior, and for the protection of retina from light-induced damage [14–17]. Differences in the activity of spin-traps and classical antioxidants (enzymatic or nonenzymatic) are that the latter may indiscriminately convert both normal oxygen and ROS molecules to water alone, leading to deep tissue hypoxia. Spin-traps react with the ROS by intercepting it before any damage is done and PBN seems to be the least toxic of nitrones [14]. PBN itself was able to act preventively and/or therapeutically in rat models of glioma, the choline deficiency liver cancer model, and the mouse APCMin/+ colon cancer model [13] and it can probably act similarly on other malignancies.

It must be stressed that the mechanism of therapeutic activity of nitrones is not yet clear. Nitrones act on a variety of pathways to change the redox state through scavenging ROS or reactive nitrogen species (ROS/RNS) but they are also involved in controlling signaling transduction and gene induction [12,18] through NO-releasing properties [19]. Nitric oxide reacts with superoxide (O_2_^–^) to form the strong oxidant peroxynitrite (ONOO–), and nitration on the three positions of tyrosine represents a major product of peroxynitrite attack on proteins that may disrupt phosphorylation that is crucial for signal transduction pathways [20].

Increase of 8-hydroxy-2′deoxyguanosine (8-OHdG), a marker of DNA oxidative damage, after experimental treatment of the kidney with carcinogen was partially prevented by PBN [21]. Importantly, 8-OHdG was correlated to methylation of 11 investigated tumor suppressor genes in the development of hepatic cancer [22]. However, DNA oxidative damage adducts may also decrease the binding capacity of DNA methyltransferases (DNMTs), leading to hypomethylation and genomic instability [23].

DNA methylation is an epigenetic mechanism that regulates gene expression [24] and, consequently, developmental processes during gestation or in the adult organism. If DNA methylation changes under the influence of the macro/microenvironment, deregulated developmental processes can lead to fetal malformations [25] or malignancy [26]. Oxidative stress represents such an important influence that has been associated with aberrant DNA methylation in reproduction [27] and carcinogenesis [28]. In carcinogenesis, oxidative stress was associated with both global hypomethylation [23,29] and hypermethylation of gene promotors, for example, involvement in myelodysplastic syndrome (MDS) [30]. Oxidative stress and DNA methylation have a common denominator: the one carbon cycle [27,31].

Necessary DNA methylation is acquired through DNMTs, and passive demethylation is acquired in the absence of DNMTs. Active demethylation proceeds through oxidative processes *via* the ten-eleven translocation methylcytosine dioxygenase (TET) enzymes [32,33] or by deamination of the amine group of 5mC by activity-induced deaminase/apolipoprotein B mRNA editing enzyme, catalytic polypeptide-like (AID/APOBEC) [34]. Aberrant hyper- or hypo-methylations are considered epimutations that can be heritable, at least in daughter cells. Unlike classical mutations, epimutations may be reversed by epigenetic therapeutics [35,36].

For example, DNA hypomethylating drug 5azaC and its deoxy-derivative, 5-aza-2′-deoxycytidine are the first-line treatment of MDSs characterized by immaturity of bone marrow cells that occurs especially in elderly patients and are also the treatment of acute myeloid leukemia [37]. 5azaC is a chemical analogue of the nucleoside cytidine that incorporates itself in both RNA and DNA, and 5-aza-2′-deoxycytidine incorporates itself in DNA, alone. By their DNA hypomethylating activity, achieved through inactivation of DNMT1, they are able to reactivate some genes, such as tumor suppressors. They also exert cytotoxic activity at higher doses [37]. DNA hypomethylating drugs are “genomic medicines” that act on the whole methylome [38] and, therefore, have numerous targets that can interact in many ways, some of which still have to be discovered and explained. Interestingly, the first global evaluation of the epigenetic effects of 5azaC on MDS bone marrow progenitor cells covering DNA methylation, repressive/permissive histone marks and gene expression, did not discover any major/consistent epigenetic changes, aside from the activation of endogenous retroviruses (ERVs) that may contribute to the clinical effects of 5azaC [39].

As mentioned earlier, proper genomic methylation is of utmost importance for normal development [40,41], whereas its aberrations caused by DNA hypomethylating drugs cause teratogenesis as a serious side-effect [1,2].

To assesses the possible beneficial effect of PBN pretreatment on embryonic development impaired by 5azaC, this research focused on the effects in offspring of pregnant dams treated on two consecutive days (days 12 and 13 of pregnancy), each time by a single dose of 5azaC. Apart from being characterized by extensive growth of embryos that may be influenced by extraneous factors, this period of development represents a sensitive time-window for induction of gross malformations, especially of the limbs because they start to develop, first the forelimb buds (day 12) and 1 day later (day 13) the hindlimb buds. Moreover, the liver starts to develop during that period and, therefore, should be susceptible to embryotoxic/teratogenic agents [42–44].

Our results suggest that the spin-trap PBN can ameliorate the most pronounced negative effects of 5azaC on the development of the offspring through scavenging of free radicals.

## Materials and Methods

### Ethical statement

All procedures on animals were conducted according to the Directive 2010/63/EU and Croatian Law on protection of experimental animals. The Ethical Committee of the School of Medicine, University of Zagreb, Croatia, approved them.

### Animals

Three-month-old female Fisher inbred strain rat dams conventionally caged, with water and standard diet ad libitum were caged overnight with males and if sperm was found in the vaginal smear in the morning of the next day, it was assigned as gestation day 0. Pregnant female rats received 5azaC (Sigma A 2385) (5 mg/kg) dissolved in phosphate-buffered saline (PBS) on days 12 and 13 of gestation by the i.p. injection. PBN (Sigma B 7263) (40 mg/kg) was injected in the tail vein on days 12 and 13 of gestation as pretreatment 1 h before 5azaC. Control dams were injected with PBS on days 12 and 13 of gestation by i.p. or i.v. injections.

### Sample isolation and processing

Pregnant females were anesthetized i.p. with 0.8 mL/kg of ketamine (Narketan^®^; Vétoquinol, Bern, Switzerland) and 0.6 mL/kg of xylazine (Xylapan^®^; Vétoquinol) on days 15 or 20 of gestation and their uteruses were isolated. Animals were sacrificed by this procedure. Embryos (day 15) and fetuses (day 20) were isolated according to the Witshi staging [42]. Embryonic and fetal crown-rump lengths (C-R length) and weights were measured. Samples were fixed in St. Marie solution (1% glacial acetic acid in 96% EtOH), dehydrated, and embedded in paraffin. Serial step sections (5 μm) were processed for routine histology or immunohistochemistry.

Resorptions and deaths in utero were recorded at day 15. The presence of malformations was determined under the dissecting microscope in fetuses at day 20. Differential staining of cartilage and bone was recorded after the alizarin red S and alcian blue staining for 4 days at 37°C, and it was further processed according to a modified Inouye method [45].

### DNA isolation

At least six limb bud samples/group were deparaffinized by using xylene (2 × 5 min) followed by incubation in 100%–95%–70% ethanol (3 min each) in water. DNA was extracted in TE buffer pH9 with 0.1 μg/μL of Proteinase K and 0.25% of Nonidet P40 at 56°C for 24 h [46]. Next, samples were heated for 10 min at 95°C to inactivate Proteinase K, spun and the supernatant was frozen at −20°C. DNA concentrations and quality were measured with the NanoDrop ND-2000 spectrophotometer (NanoDrop Technologies, Wilmington, DE).

### Bisulfite conversion and polymerase chain reaction

One thousand nanograms of unpurified isolated genomic DNA was used for bisulfite conversion by EpiTect Plus DNA Bisulfite Kit (#59124; Qiagen), which includes a clean-up step with no necessity for prior purification of DNA. PyroMark PCR Kit (#978703; Qiagen) was used for polymerase chain reaction (PCR) amplification in the following conditions: 95°C for 2 min, 43°C for 90 s, and 72°C for 60 s for 40 cycles with the following PCR primers: forward primer: 5′-GGGTTGGGGATTTAG-3′ and biotinylated reverse primer: 5′-AACCCAAAACCTTA-3′.

### Global methylation analysis

Global methylation was measured by pyrosequencing. All the steps were performed as recommended by the manufacturer (Qiagen). Pyromark Q24 Advanced System with PyroMark Q24 CpG Advanced Reagents (#970922; Qiagen) was used for the pyrosequencing reaction; 5′-GGGGATTTAGTTTAGTGGT-3′ was the sequencing primer for the rat ID element [47]. DNA methylation data were obtained and analyzed by the PyroMark Q24 Advanced Software.

### Immunohistochemistry

Primary mouse monoclonal antibody against proliferating cell nuclear antigen [monoclonal mouse anti-proliferating cell nuclear antigen (PCNA), clone PC 10, M0879 (Dako, Glostrup, Denmark)] was used (1:50) for the detection of proliferating cells. The antibody was incubated overnight at 4°C. The standard negative control reagent (V1617; Dako) was used to inhibit the appearance of endogenous signal. PCNA expression was visualized by using the LSAB2 System-HRP for use on Rat Specimens Kit (K0609; Dako) according to the manufacturer's instructions. Oxidative stress was detected by 8-OHdG (1:500, sc-66036) and nitrotyrosine (1:50, sc-32757), both from Santa Cruz Biotechnology, Inc. (Dallas, TX). The antibodies were incubated overnight at 4°C. Sections were treated with Dako REAL™ EnVision™/HRP, Rabbit/Mouse reagent (K5007; Dako; Agilent Technologies, Inc., Santa Clara, CA); 3,3′-diaminobenzidine-tetrahydrochloride (DAB) was used for signal staining, and hematoxylin was used for counterstaining.

### Oxidative stress analysis

The expression of 8-OHdG and nitrotyrosine in single cells of embryonal tissues was used to calculate the histological score (H-score) [48,49]. In three specimens per group, the staining intensity (0, 1+, 2+, or 3+) was determined for each cell in a field of 500 cells. A score 0–300 was assigned to each specimen, based on multiplication of the percentage of cells with different staining intensity within the same field. The discriminatory threshold was set at 50, and samples were considered low (H-score <50) or high (≥50) [50].

### Quantitative stereological analysis

Binocular light microscope “Nikon Alphaphot” × 400 for stereological analysis was used along with the Weibel's 42-points multipurpose test system (M42) [51]. The overall length of test lines (*L_t_*) was 1.008 mm, and the area tested (*A_t_*) was 0.0837 mm^2^ for each analyzed microscopic field. Sample size (no. of fields to be tested) was determined after analyzing samples on 10 fields with a 95% confidence interval by using the formula “*n*” = (200/*y* × *s*/*x*)^2^, where “*n*” marks the number of fields that need to be analyzed (at least 30 fields per sample), *x* is the arithmetic mean of the orientation sample, *s* is the standard deviation (SD) of the orientation sample, and *y* is the allowed variation from the arithmetic mean [52]. PCNA-positive cells were counted in samples and the numerical density (*N_v_*) was determined by the formula *N_v_* = *N*/*A_t_* × *D*, where *N* is the number of PCNA-positive cells on the tested area. *D* is the mean tangential nucleus diameter calculated by light microscopy for 100 cells (for hepatocyte nuclei *D* = 0.0146 mm, for chondrocyte nuclei *D* = 0.0147, and for limb bud cell nuclei *D* = 0.076 mm), and *A_t_* is 0.0837 mm^2^. The 3D Ellipse stereological program was used to measure PCNA-positive nucleus diameter.

### Apoptotic index

Hematoxylin–eosin-stained specimens were used to measure the apoptotic index. Ten microscopic fields per sample were randomly selected. The histological analysis was performed at a high magnification of 1,000 × by counting apoptotic figures—cells with typical apoptotic morphology (condensed chromatin, cell shrinkage, fragmented nucleus, eosinophilic cytoplasm). The apoptotic index was determined as the number of apoptotic cells per 100 cells in a field [53]. Three specimens from different dams per group were analyzed.

### Statistical methods/data analysis

The primary outcome measures were embryonic weights and C-R lengths differences between the control group and groups treated with 5azaC and/or PBN. Secondary outcome measures were the differences in the presence of PCNA-positive cells, differences in malformation rates between the different groups and the DNA methylation pattern between the treated groups and controls. Statistical evaluation of weights and lengths, PCNA numerical density, H-score, apoptotic indices, and global DNA methylation levels within groups were evaluated by using the Student's *t*-test, Mann–Whitney test, ANOVA with Holm–Sidak's multiple-comparison post hoc test, or Kruskal–Wallis test with Dunn's multiple-comparison post hoc test. Coefficient of variation was expressed as SD/mean × 100. Survival and malformation comparison were done by *χ*^2^ analysis. Before analyses, the descriptive statistics was done by the D'Agostino and Pearson test. GraphPad Prism software (version 6.0, GraphPad Software, Inc., San Diego, CA) was used for data analysis. Statistical significance was set at *P* ≤ 0.05.

## Results

### Survival

After treatment with PBN, 5azaC, or PBN and 1 h later with 5azaC on days 12 and 13 of pregnancy, all rat dams survived until day 20 of pregnancy with no visible side-effects on inspection.

On day 15 of gestation, the survival, intrauterine deaths, and resorption were assessed. Embryos assigned to the intrauterine death group were well formed although smaller and paler than controls, whereas those that were assigned to the resorption group were amorphic and much smaller. PBN alone had no impact on survival, which was comparable to the 100% survival rate in the control group. Survival in the group treated with 5azaC was poor (59%), resulting in intrauterine deaths and resorption. When PBN was administered 1 h before 5azaC, the survival rate was significantly better (85.5%) than affected by the treatment with 5azaC (Table 1).

**Table 1.** Survival of Embryos Previously Treated with 5-Azacytidine and/or *N*-Tert-Butyl-α-Phenylnitron

	*No. of embryos*		*Survival*		*Resorptions*		*Intrauterine deaths*	
	N	*%*	N	*%*	N	*%*	N	*%*
Control	43	100	43^a,b^	100	0	0	0	0
PBN (40 mg/kg)	72	100	72	100	0	0	0	0
5azaC (5 mg/kg)	64	100	38^b,c^	59.4	15	39.47	11	28.9
5azaC (5 mg/kg) + PBN (40 mg/kg)	83	100	71^a,c^	85.5	10	14.08	2	2.8

^a^ *χ*^2^ = 6.8713; *P* = 0.008759216.

^b^ *χ*^2^ = 23.076; *P* = 0.0000001.

^c^ *χ*^2^ = 12.909; *P* = 0.0003.

5azaC, 5-azacytidine; PBN, *N*-tert-butyl-α-phenylnitron.

### Overall growth

**FIG. 1.**
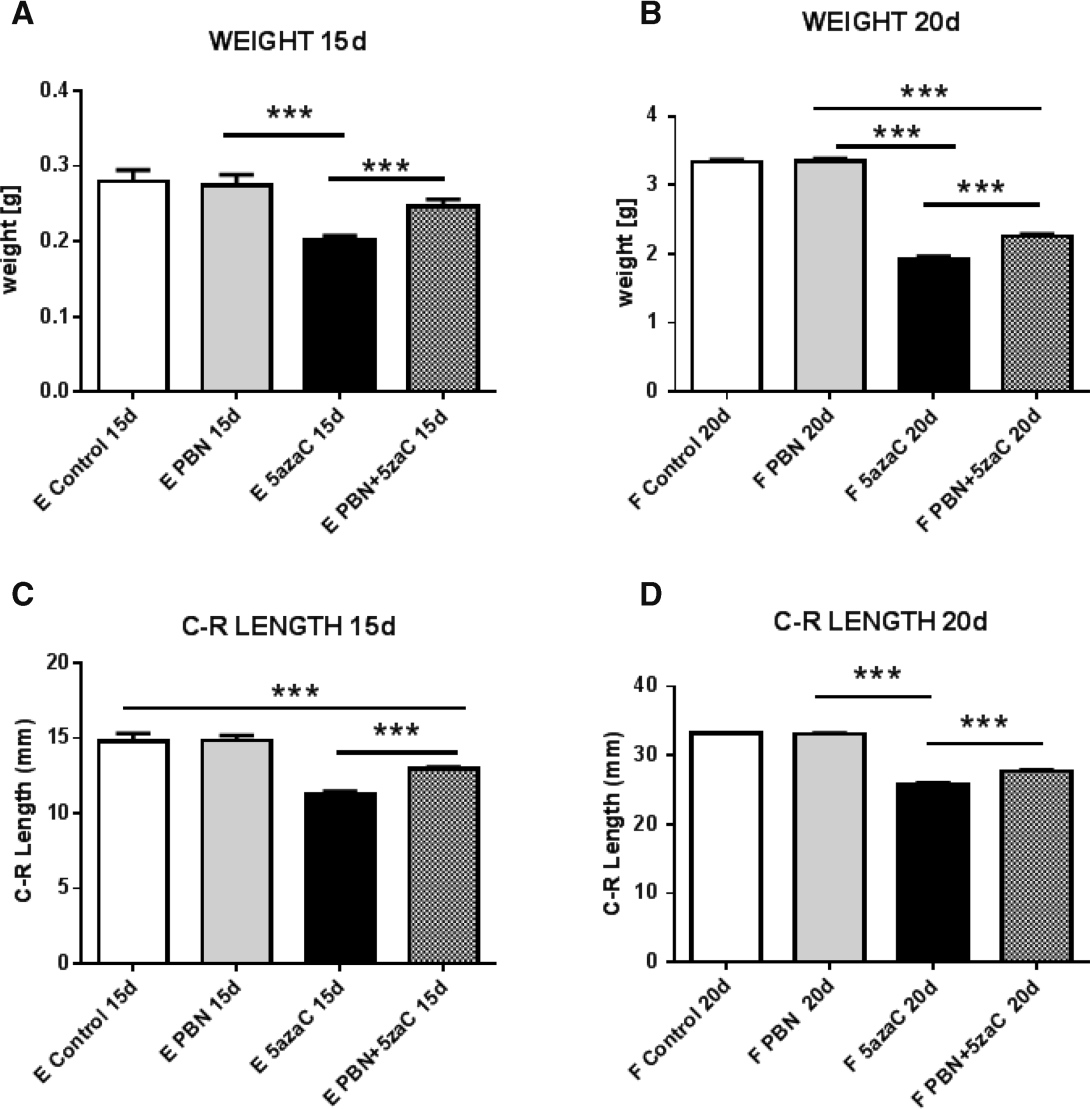
Overall growth of embryos **(A, C)** and fetuses **(B, D)** previously treated with 5azaC, PBN or pretreated with PBN and then with 5azaC was measured by body weight or by C-R length. Values represent mean ± SEM. ANOVA or Kruskall–Wallis and Mann–Whitney test. ***P < 0.001. 5azaC, 5-azacytidine; C-R length, crown-rump length; PBN, *N*-tert-butyl-α-phenylnitron; SEM, standard error of the mean.

To assess whether overall growth was affected by treatments, both the weight and C-R length measurements were assessed at the embryonic and more advanced fetal stage of development. All surviving offspring were able to grow from day 15 until day 20 of the pregnancy, shown by substantially increased weights and C-R lengths (Fig. 1).

Embryos derived from dams treated with PBN alone weighted as untreated controls and weighted significantly more than 5azaC-treated dams. Embryos from PBN-pretreated dams weighted significantly more than those from of 5azaC-treated dams, with no statistical difference in comparison to the control (Fig. 1A). Direct comparison of the group of fetuses from dams treated with 5azaC only and the group pretreated with PBN showed that fetuses in the pretreated group were significantly heavier than those treated only with 5azaC (Fig. 1B).

The C-R length of embryos and fetuses from dams treated with PBN showed no difference compared with the C-R length in the control groups. Embryos from dams treated only with 5azaC or pretreated with PBN were statistically smaller than controls. A direct comparison of two groups treated with 5azaC showed that pretreatment with PBN leads to a significant increase in size of the embryos (*P* < 0.001) (Fig. 1C, D).

### Malformations

**FIG. 2.**
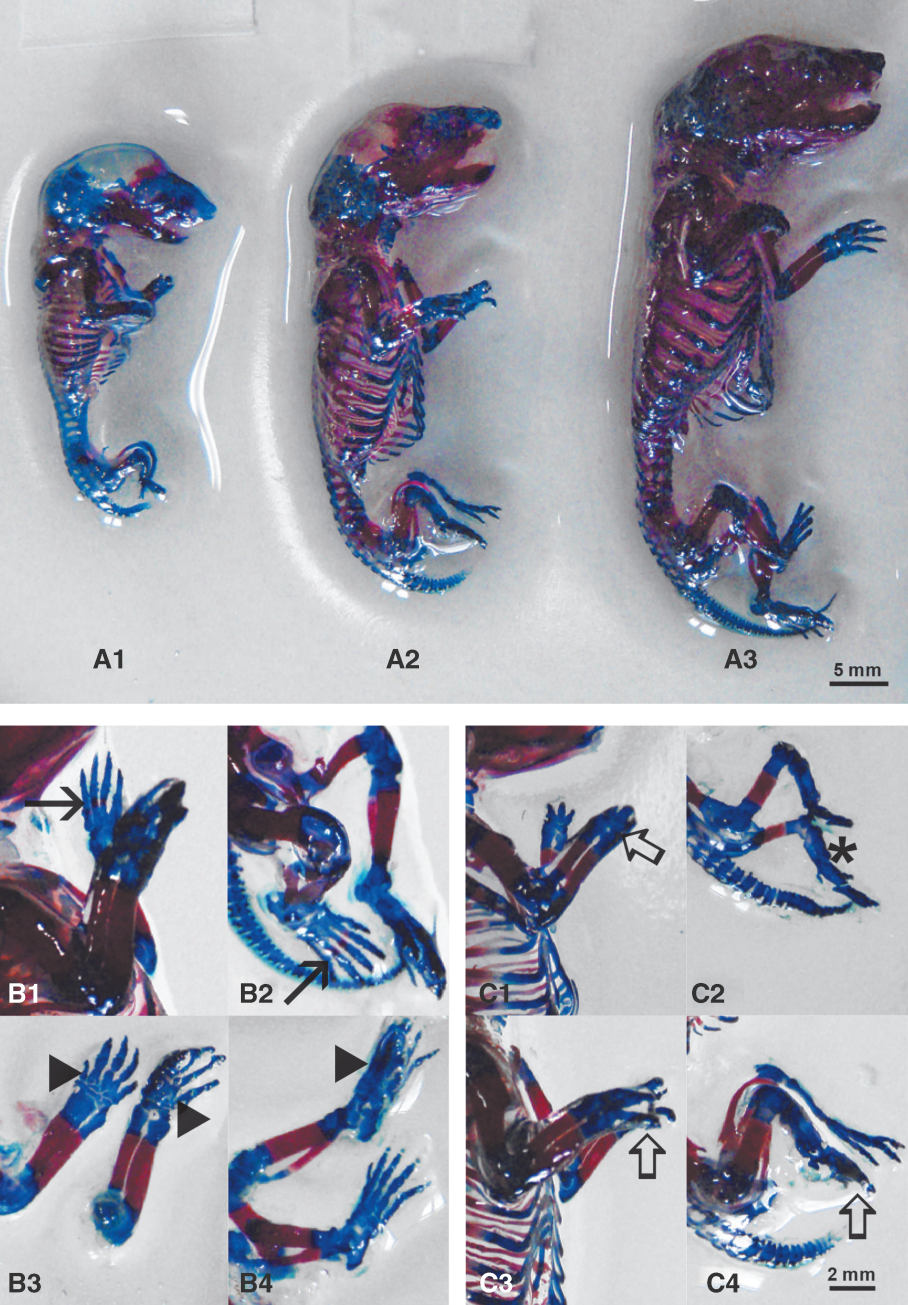
Skeletal development and malformations in fetuses from dams previously treated with 5azaC and/or PBN: **(A1)** 5azaC-treated, **(A2)** PBN-pretreated and 5azaC treated, **(A3)** PBN-treated. Ossification: **(B1)** in control forelimb (*arrow*), **(B2)** in control hindlimb (*arrow*), **(B3)** PBN-treated forelimbs—missing ossification (*arrowhead*) and **(B4)** PBN-treated hindlimbs. **(C1)** forelimbs of fetuses from dams treated with 5azaC, oligodactyly (*thick arrow*), **(C2)** hindlimbs of fetuses from dams treated with 5azaC, adactyly (*asterisk*). **(C3)** forelimbs and **(C4)** hindlimbs of fetuses from dams treated with PBN and 5azaC, oligodactyly (*thick arrow*). Alizarin red S, Alcian blue, Inouye method. Color images are available online.

To determine whether fetuses exhibited any specific malformations, we inspected them under the dissecting microscope. The group of fetuses from dams treated with PBN alone showed no signs of malformations (Fig. 2A), the same as untreated controls (not shown). The only difference noted after PBN treatment of dams was the absence of palmar and plantar ossification nuclei (Fig. 2B). All fetuses from dams treated with 5azaC had malformations of forelimbs and hindlimbs (adactyly, oligodactyly) (Fig. 2C). Malformations were also present in 5azaC-treated fetuses from dams pretreated with PBN, and there was no statistical difference in the overall number of malformed fetuses as well as the number of limb malformations between the two groups. However, pretreatment of dams with PBN before 5azaC treatment significantly reduced the number of adactyly in the fetuses and significantly increased the number of fetal oligodactyly (Table 2).

**Table 2.** Malformations in Fore- and Hindlimb Buds of Fetuses Derived from Dams Previously Treated with 5-Azacytidine and/or *N*-Tert-Butyl-α-Phenylnitron

	*5azaC (5 mg/kg)*		*5azaC (5 mg/kg) + PBN (40 mg/kg)*	
	N	*%*	N	*%*
Malformed/isolated fetuses	38/38	100	69/71	97.18
Forelimbs				
Malformed/isolated fetuses	38/38	100	69/71	97.18
Adactyly	36^a^	94.74	54^a^	76.06
Oligodactyly	9^b^	23.68	47^b^	66.20
Hindlimbs				
Malformed/isolated fetuses	38/38	100	69/71	97.18
Adactyly	34^c^	89.47	36^c^	50.7
Oligodactyly	12^d^	31.58	55^d^	77.46

^a^ *χ*^2^ = 6.00135773; *P* < 0.02.

^b^ *χ*^2^ = 17.9080167; *P* < 0.001.

^c^ *χ*^2^ = 16.1914325; *P* < 0.001.

^d^ *χ*^2^ = 22.0040057; *P* < 0.001.

### DNA methylation in limb buds

**FIG. 3.**
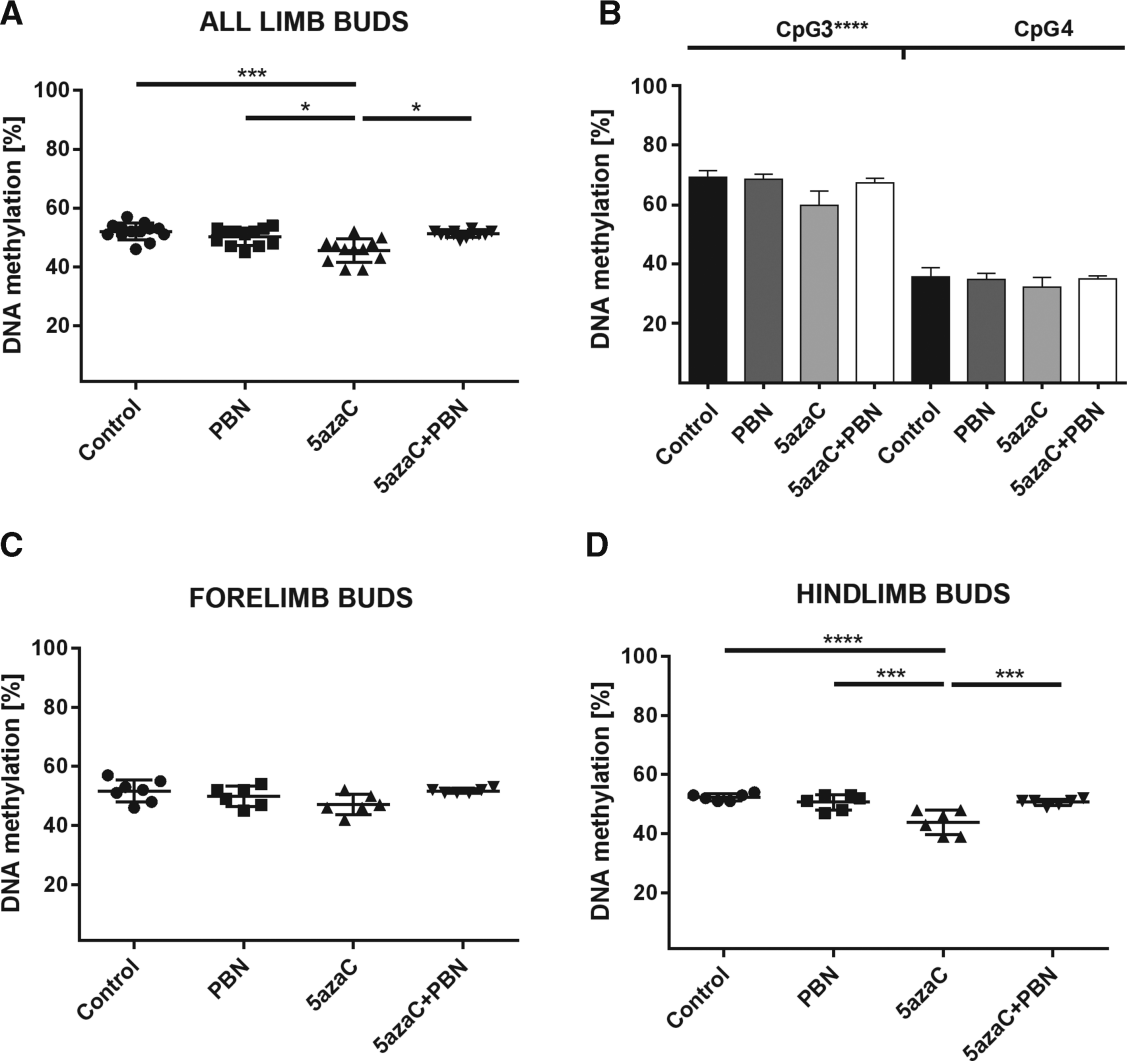
Global DNA methylation in limb buds of embryos from dams previously treated with 5azaC and/or PBN. **(A)** 5azaC induced strong global DNA demethylation in limb buds that could be prevented by PBN. ANOVA. *P < 0.05, ***P < 0.001. **(B)** Statistically significant global demethylation was detected in CpG3 but not in CpG4 of the chosen ID element. ANOVA. ****P < 0.0001. **(C)** Methylation of forelimb buds and **(D)** hindlimb buds. Statistically significant decrease in global methylation appears only in the hindlimb buds. Kruskall–Wallis; Dunn's post hoc test. ***P < 0.001, ****P < 0.0001. ID, identifier.

To determine whether the DNA demethylating agent 5azaC impaired DNA methylation in limb buds as the precursors of malformed limbs, we assessed global DNA methylation. That was done by using identifier (ID) element previously recommended for routine analysis of the global DNA methylation changes in rats for pharmaceutical treatment [47]. Our analysis was performed by bisulfite pyrosequencing assay that has been strongly recommended for the best all-round performance among other contemporary DNA methylation assays and is appropriate for sequencing short sequences [54] such as widely spread rat ID elements [47]. In embryonic limb buds from 5azaC-treated dams, statistically significant global DNA demethylating effect was found compared with the embryos from control group dams and PBN-treated dams (Fig. 3A). In limb buds of embryos from PBN-pretreated dams, DNA methylation levels were as in controls, suggesting that PBN prevented 5azaC-induced global demethylation in the developing limb buds. In addition, we discovered that DNA demethylation was significant in CpG3 of the ID element that was previously verified by whole genome amplification and 5azaC treatment in a cell line and not in CpG4 that was not previously verified as responsive to 5azaC treatment (Fig. 3B) [47].

However, when global DNA methylation was analyzed separately in fore- and hindlimb buds, statistically significant changes in global DNA methylation appeared to be induced in hindlimb buds only (Fig. 3C, D). Forelimb buds that are developmentally more advanced than hindlimb buds [55] were not as significantly affected by the treatment of their dams with 5azaC. Therefore, the intensity of 5azaC demethylating effect depended on the developmental stage of the limb buds.

### Cell proliferation

**FIG. 4.**
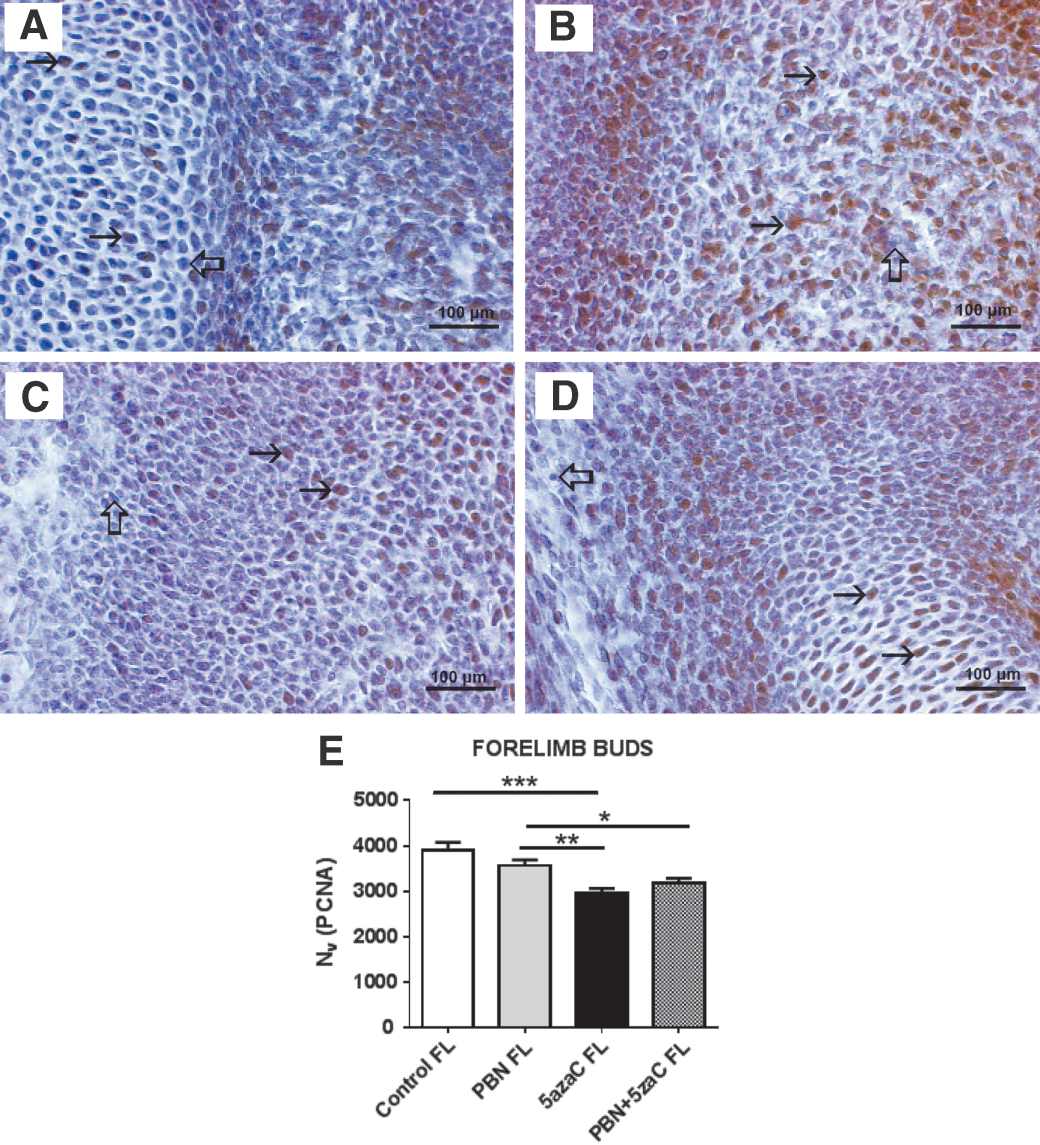
PCNA expression (*arrows*) in forelimb buds of embryos derived from dams previously treated with 5azaC and/or PBN, detected by immunohistochemistry: **(A)** control; **(B)** PBN-treated; **(C)** 5azaC-treated; **(D)** PBN-pretreated and 5azaC-treated forelimb bud. DAB, hematoxylin counterstain. *Thick arrows* point to internal negative controls. Quantification of PCNA-positive signals **(E)** by the stereological measurement of numerical density (*N_v_*). *N_v_* values (columns) represent mean ± SEM. ANOVA, Holm–Sidak's multiple-comparison post hoc test. *P < 0.05, **P < 0.001, and ***P < 0.0001. DAB, diaminobenzidine-tetrahydrochloride; PCNA, proliferating cell nuclear antigen. Color images are available online.

**FIG. 5.**
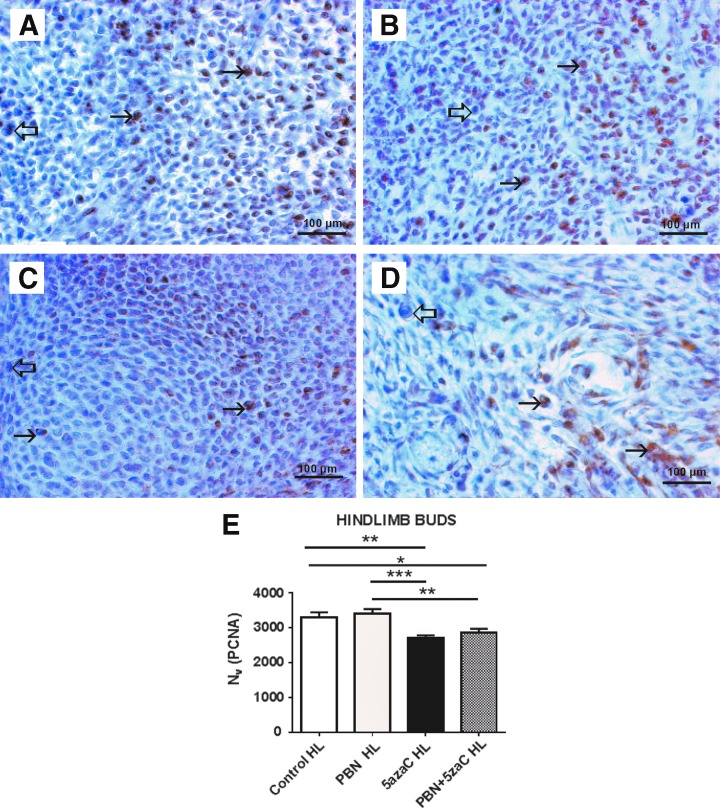
PCNA expression (*arrows*) in hindlimb buds of embryos from dams previously treated with 5azaC and/or PBN, detected by immunohistochemistry: **(A)** control; **(B)** PBN-treated; **(C)** 5azaC-treated; **(D)** PBN-pretreated and 5azaC-treated forelimb bud. DAB, hematoxylin counterstain. *Thick arrows* point to internal negative controls. Quantification of PCNA-positive signals **(E)** by the stereological measurement of numerical density (*N_v_*). *N_v_* values (columns) represent mean ± SEM. ANOVA, Holm–Sidak's multiple-comparison post hoc test. *P < 0.05, **P < 0.001, and ***P < 0.0001. Color images are available online.

**FIG. 6.**
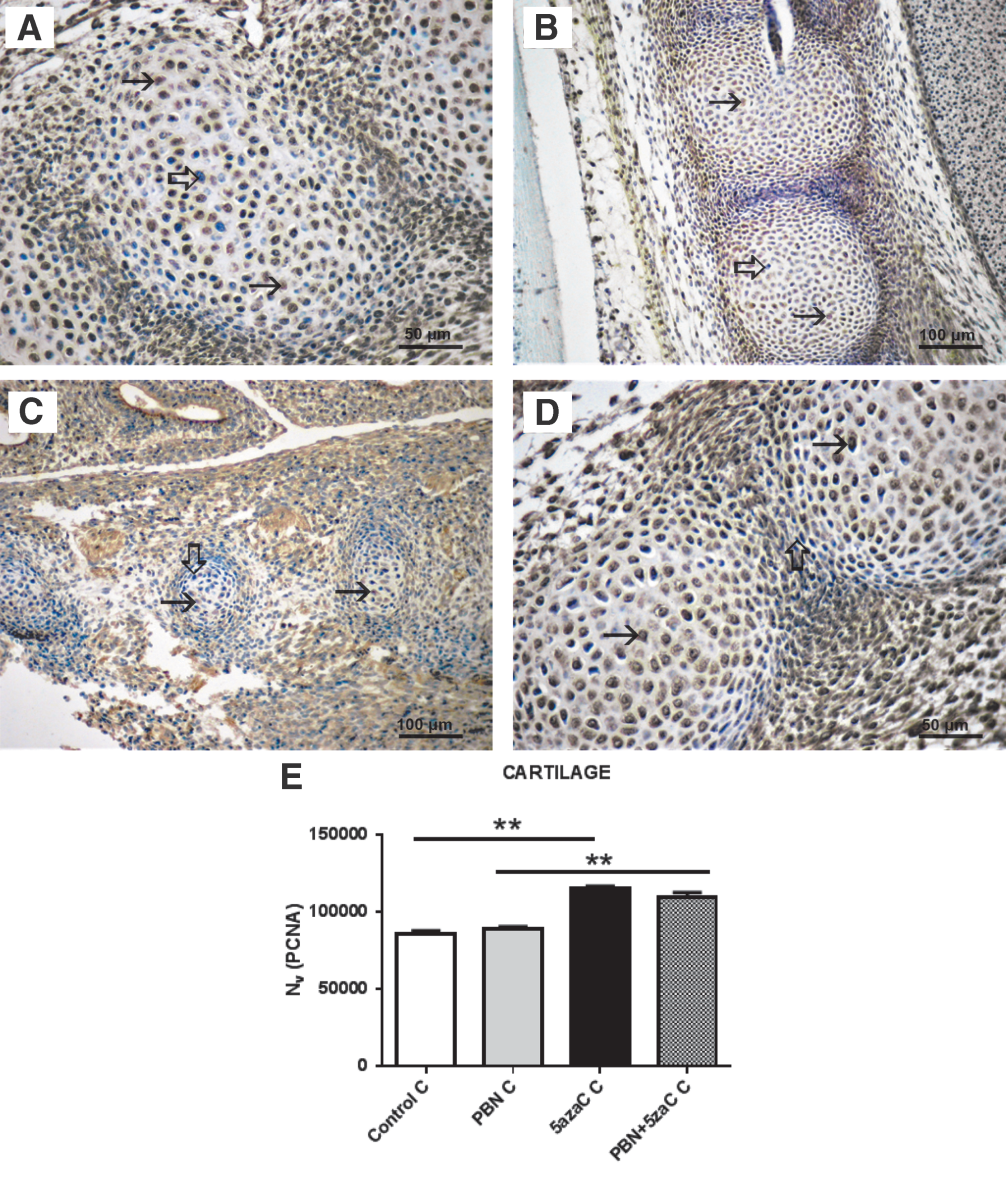
PCNA expression (*arrows*) in cartilage of embryos derived from dams previously treated with 5azaC and/or PBN, detected by immunohistochemistry: **(A)** control; **(B)** PBN-treated; **(C)** PBN-pretreated and 5azaC-treated; **(D)** 5azaC-treated cartilage. DAB, hematoxylin counterstain. *Thick arrows* point to internal negative controls. Quantification of PCNA-positive signals **(E)** by the stereological measurement of numerical density (*N_v_*). *N_v_* values (columns) represent mean ± SEM. Kruskall–Wallis; Dunn's post hoc test. **P < 0.001. Color images are available online.

**FIG. 7.**
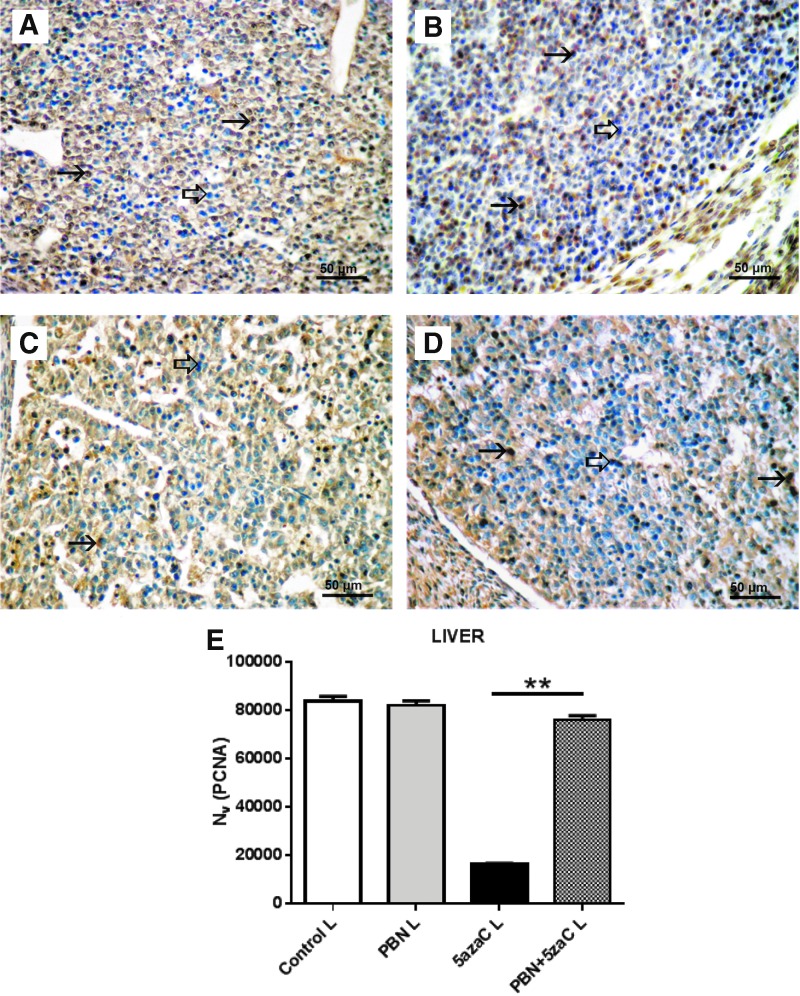
PCNA expression (*arrows*) in liver of embryos derived from dams previously treated with 5azaC and/or PBN, detected by immunohistochemistry: **(A)** control; **(B)** PBN-treated; **(C)** PBN-pretreated and 5azaC-treated; **(D)** 5azaC-treated cartilage. DAB, hematoxylin counterstain. *Thick arrows* point to internal negative controls. Quantification of PCNA-positive signals **(E)** by the stereological measurement of numerical density (*N_v_*). *N_v_* values (columns) represent mean ± SEM. Kruskall–Wallis; Dunn's post hoc test. **P < 0.001. Color images are available online.

To determine whether the treatments of dams affected cell proliferation in their embryos, we again assessed PCNA expression at the single-cell level and positive signals were quantified by numerical density (*N_v_*) (Figs. 4–7). First, we assessed cell proliferation within the embryonic fore- (Fig. 4) and hindlimb (Fig. 5) buds that subsequently developed into malformed limbs in fetuses. There was no significant difference between the numerical density (*N_v_*) of PCNA when their dams were treated only with PBN or sham-treated controls. Significantly lower proliferation (*N_v_*) in comparison to controls and PBN-treated embryos was found in limb buds treated only with 5azaC and those pretreated with PBN before 5azaC treatment (Figs. 4 and 5). Therefore, pretreatment of pregnant dams with PBN did not affect proliferation in limb buds of the 5azaC-treated dams' embryos.

To investigate whether other embryonic tissues responded in the same way to the treatment of dams as the developing limb buds, proliferation was assessed also in the cartilage of the vertebrae (Fig. 6) and in the liver (Fig. 7).

Contrary to the negative effect of the 5azaC detected in limb buds, PCNA expression in cartilage of the vertebrae was highest in both groups of embryos from dams treated with 5azaC, and it was significantly lower in all PBN-treated dams and controls (Fig. 6). Therefore, pretreatment of dams with PBN did not influence high cell proliferation in the embryonic cartilage.

Similarly as in limb buds, the numerical density (*N_v_*) of PCNA expression in the liver of embryos from dams treated with 5azaC exhibited a statistically significant lower *N_v_* of PCNA whereas in PBN pretreatment it did not differ from the PBN-treated dams or controls. Therefore, PBN pretreatment was able to abolish the negative impact of 5azaC on liver cells proliferation (Fig. 7).

### Apoptosis

**FIG. 8.**
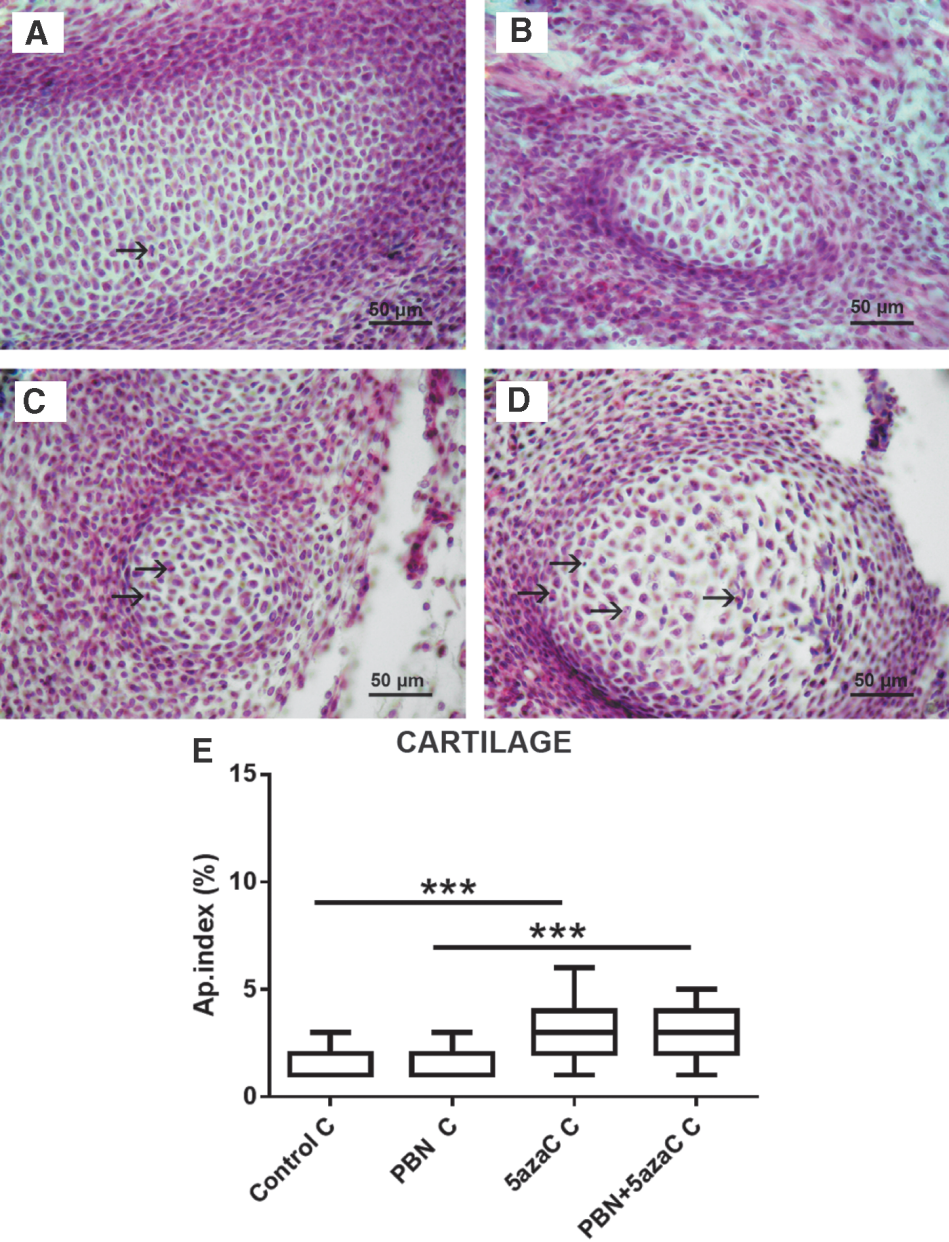
Apoptosis in the cartilage of embryos derived from dams, previously treated with 5azaC and/or PBN. Apoptotic figures (*arrows*) in **(A)** control; **(B)** PBN-treated; **(C)** PBN-pretreated and 5azaC-treated; **(D)** 5azaC-treated cartilage. Hematoxylin–eosin. Apoptotic index in embryonic cartilage **(E)**. *Boxplot* shows apoptotic index distribution (*box* denotes median, and *whiskers* denote the smallest and the largest observation). Kruskall–Wallis ANOVA; post hoc Dunn's test. ***P < 0.0001. Color images are available online.

**FIG. 9.**
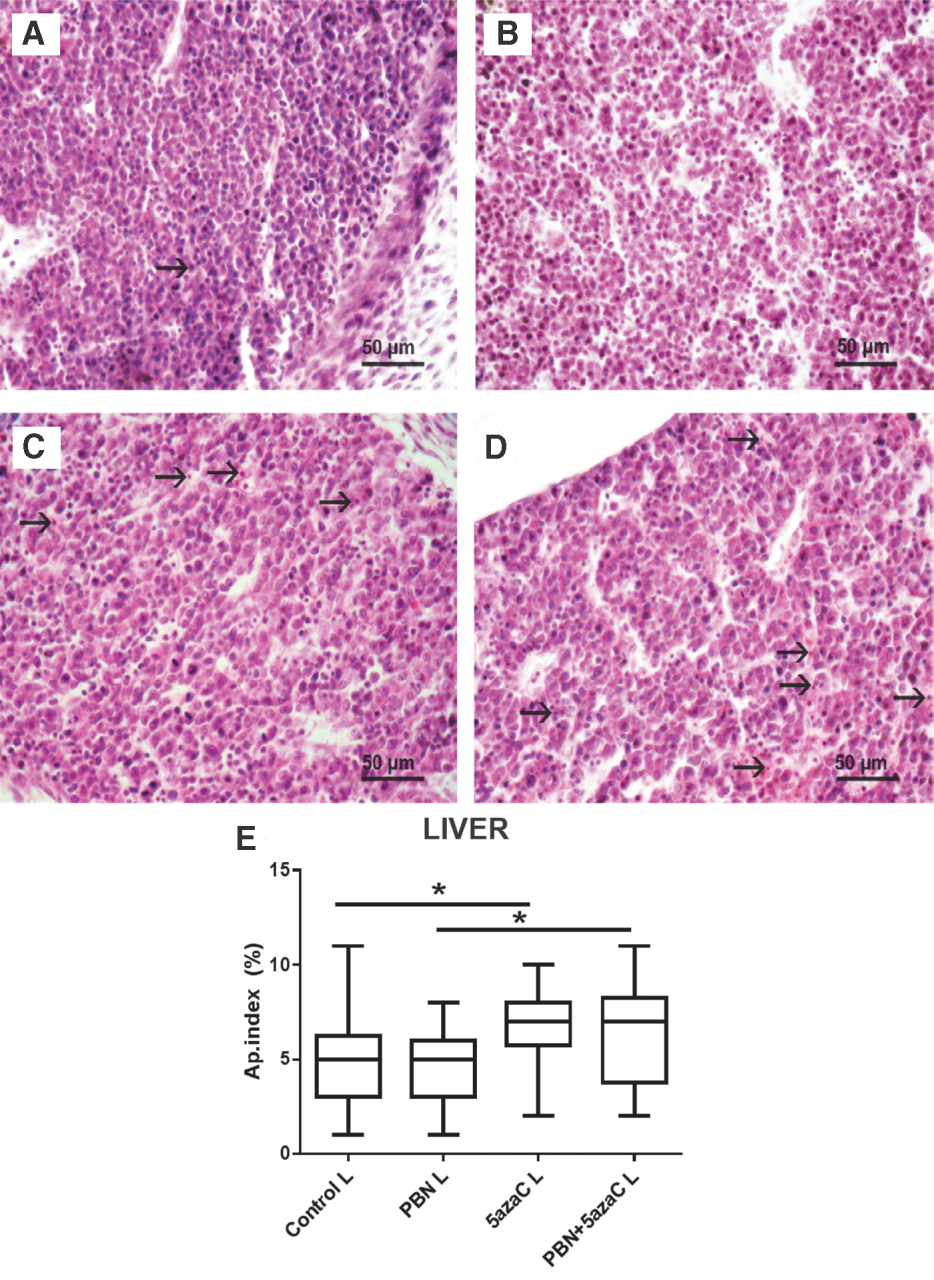
Apoptosis in liver tissue of embryos derived from dams, previously treated with 5azaC and/or PBN. Apoptotic figures (*arrows*) in **(A)** control; **(B)** PBN-treated; **(C)** PBN-pretreated and 5azaC-treated; **(D)** 5azaC-treated liver tissue. Hematoxylin–eosin. Apoptotic index in embryonic liver **(E)**. *Boxplot* shows apoptotic index distribution (*box* denotes median, and *whiskers* denote the smallest and the largest observation). Kruskall–Wallis ANOVA; post hoc Dunn's test. *P < 0.05. Color images are available online.

Apoptosis was detected in cartilage of the vertebrae (Fig. 8) and in embryonic liver (Fig. 9). A significantly increased apoptotic index in the embryonic liver and cartilage in both groups of embryos from dams treated with 5azaC in comparison to PBN treatment was determined (Figs. 8 and 9). Therefore, PBN pretreatment influenced the high level of apoptosis neither in the liver nor in the well-differentiated embryonic cartilage.

### Oxidative stress

**FIG. 10.**
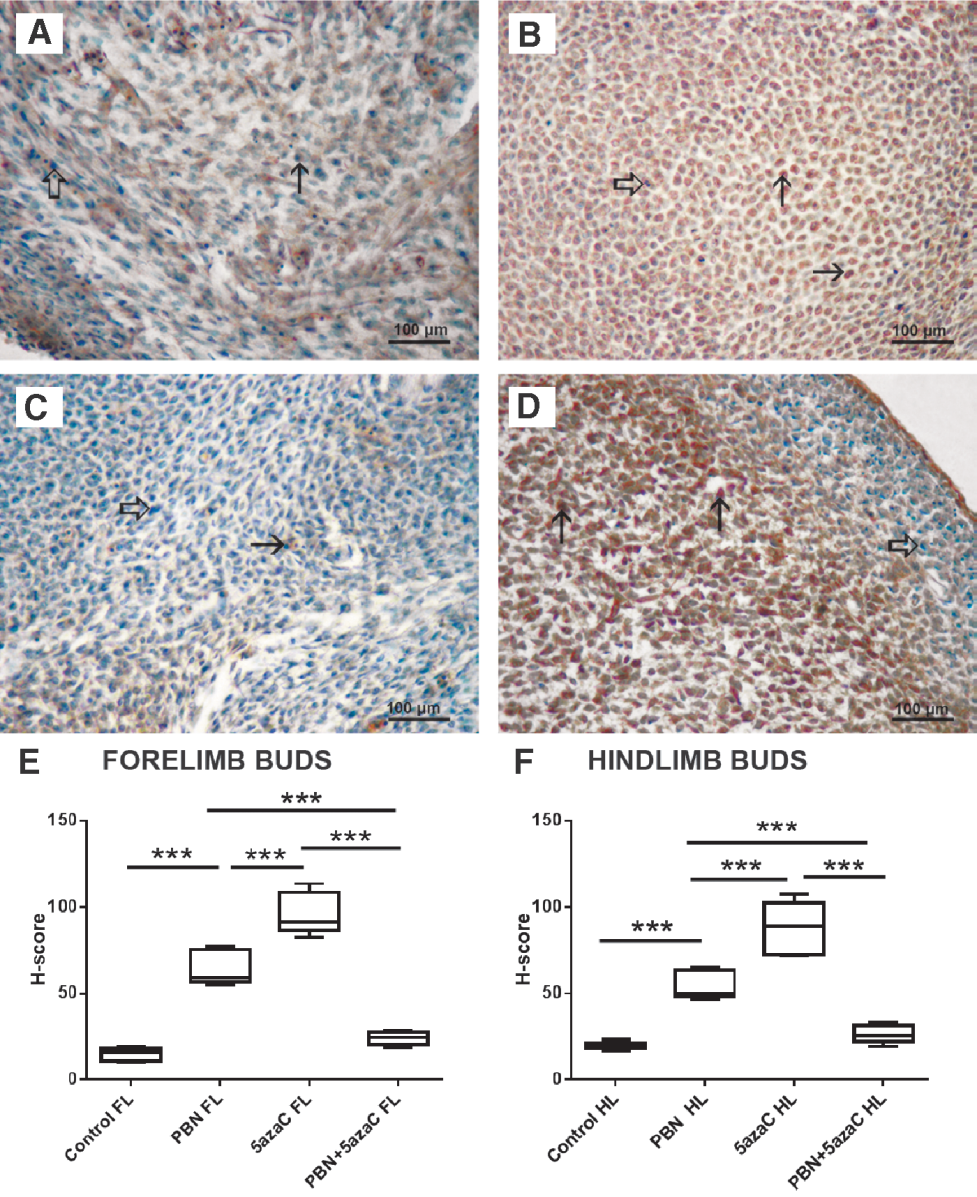
8-OHdG expression (*arrows*) in fore- and hindlimb buds of embryos derived from dams previously treated with 5azaC and/or PBN, detected by immunohistochemistry: **(A)** forelimb bud, control; **(B)** forelimb bud, 5azaC; **(C)** hindlimb bud, control; **(D)** hindlimb bud, 5azaC. DAB, hematoxylin counterstain. *Thick arrows* point to internal negative control signal. *Boxplot* shows H-score distribution in fore- **(E)** and hindlimb buds **(F)** (*box* denotes median, and *whiskers* denote the smallest and the largest observation). ANOVA, Holm–Sidak's multiple-comparison post hoc test. ***P < 0.0001. Color images are available online.

**FIG. 11.**
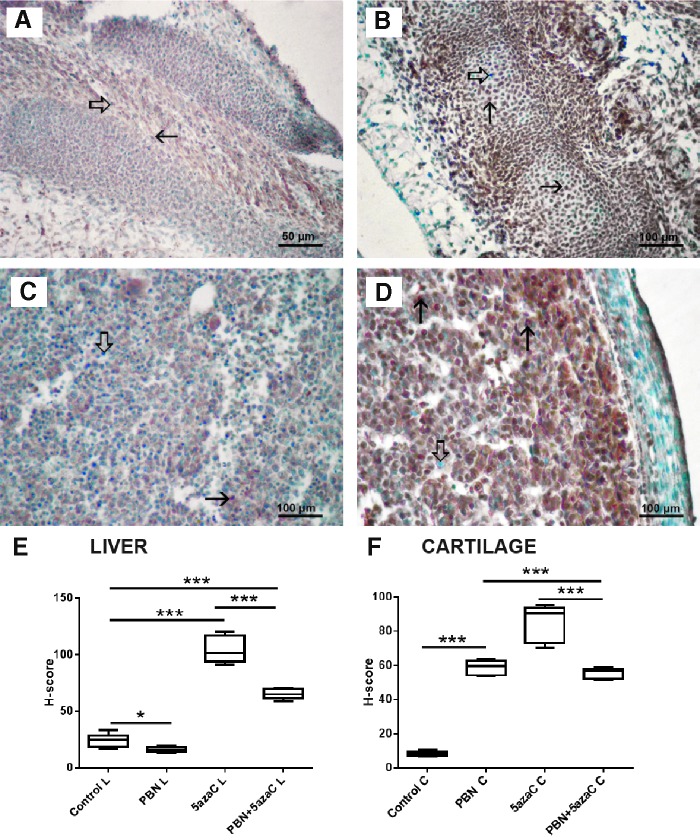
8-OHdG expression (*arrows*) in liver and cartilage buds of embryos derived from dams previously treated with 5azaC and/or PBN, detected by immunohistochemistry: **(A)** cartilage, control; **(B)** cartilage, 5azaC; **(C)** liver, control; **(D)** liver, 5azaC. DAB, hematoxylin counterstain. *Thick arrows* point to internal negative control signal. H-score in cartilage **(E)** and liver **(F)**. *Boxplot* shows apoptotic index distribution (*box* denotes median, and *whiskers* denote the smallest and the largest observation). ANOVA, Holm–Sidak's multiple-comparison post hoc test. ***P < 0.0001. Color images are available online.

**FIG. 12.**
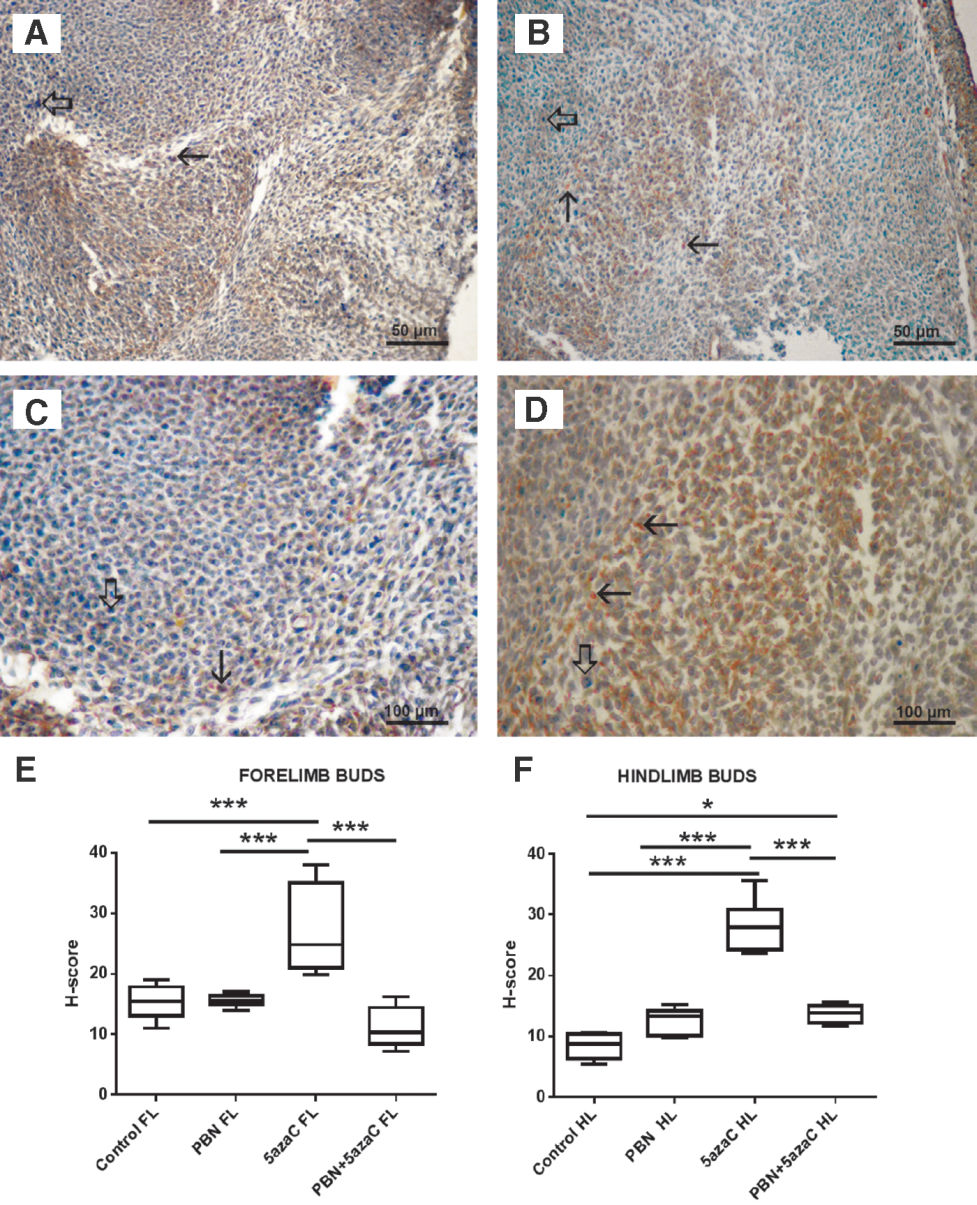
Nitrotyrosine expression (*arrows*) in fore- and hindlimb buds of embryos derived from dams previously treated with 5azaC and/or PBN, detected by immunohistochemistry: **(A)** forelimb bud, control; **(B)** forelimb bud, 5azaC; **(C)** hindlimb bud, control; **(D)** hindlimb bud, 5azaC. DAB, hematoxylin counterstain. *Thick arrows* point to internal negative control signal. H-score in fore- **(E)** and hindlimb buds **(F)**. *Boxplot* shows apoptotic index distribution (*box* denotes median, and *whiskers* denote the smallest and the largest observation). ANOVA, Holm–Sidak's multiple-comparison post hoc test. *P < 0.05, and ***P < 0.0001. Color images are available online.

**FIG. 13.**
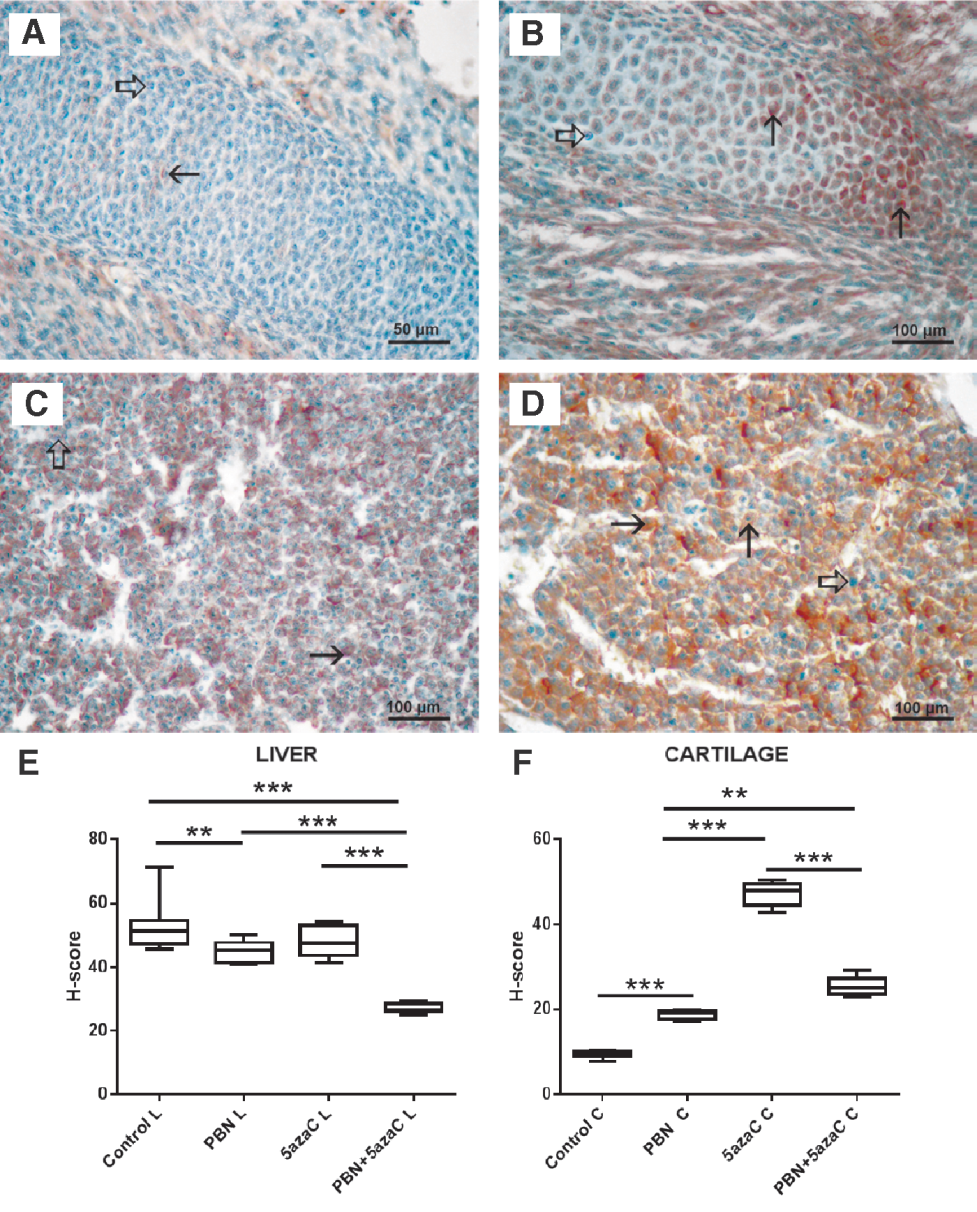
Nitrotyrosine expression (*arrows*) in cartilage and liver of embryos derived from dams previously treated with 5azaC and/or PBN, detected by immunohistochemistry: **(A)** cartilage, control; **(B)** cartilage, 5azaC; **(C)** liver, control; **(D)** liver, 5azaC. DAB, hematoxylin counterstain. *Thick arrows* point to internal negative control signal. H-score in fore- **(E)** and hindlimb buds **(F)**. *Boxplot* shows apoptotic index distribution (*box* denotes median, and *whiskers* denote the smallest and the largest observation). ANOVA, Holm–Sidak's multiple-comparison post hoc test. **P < 0.001, ***P < 0.0001. Color images are available online.

To determine whether oxidative stress took place in tissues of embryos after treatment of gestating dams with 5azaC with or without PBN pretreatment, we quantified expression of the nuclear oxidative stress-related marker 8-OHdG (Figs. 10 and 11) and the cytoplasmic oxidative stress marker nitrotyrosine (Figs. 12 and 13) in fore- and hindlimbs, cartilage, and liver.

5azaC treatment, in comparison to controls, always significantly elevated 8-OHdG expression level. Pretreatment with PBN always significantly diminished the 8-OHdG expression level in comparison to 5azaC (Figs. 10 and 11).

5azaC treatment, in comparison to controls, always (except in the liver) significantly elevated the expression of nitrotyrosine whereas PBN pretreatment always diminished nitrotyrosine expression level in comparison to 5azaC (Figs. 12 and 13).

Therefore, pretreatment of dams with PBN almost always diminished ROS activity elevated by 5azaC in tissues of their offspring.

Interestingly, PBN alone significantly diminished nitrotyrosine expression in the liver and elevated its expression in the cartilage in comparison to controls. Moreover, PBN alone was able to significantly increase 8-OHdG expression in limb buds and cartilage in comparison to the controls, although that was significantly less than with 5azaC and it diminished 8-OHdG expression in the liver. Therefore it seems that PBN alone was able to influence ROS/RNS.

## Discussion

Our results have shown for the first time that pretreatment with a nitrone significantly ameliorated the major negative impact of the DNA hypomethylating drug 5azaC on the offspring of mothers treated only twice during gestation, because a statistically significant improvement in the survival and the gross phenotype, including overall growth and limb malformations, was detected with PBN pretreatment. We confirmed that 5azaC activated ROS, as was previously published, for example, for malignant cells [56] and that PBN diminished that activity in accordance with results obtained with other teratogens [27,28]. On the other hand, 5azaC sometimes enhanced antioxidant activity of the organism, such as in lipopolysaccharide-induced acute respiratory distress in mice [57] or during therapy of MDS by induction of the antioxidant pathway Nrf2 [58]. Therefore, the impact of 5azaC on oxidative stress probably depends on a complex biological/pathophysiological context [59,60]. That is also true for PBN that in our experiments alone sometimes even enhanced oxidative stress markers in the limb buds and cartilage, although that seemed to have only a minor impact on their ossification and neither significantly changed their overall growth nor induced major limb malformations. In comparison with our previous research where pretreatment with ASA abolished limb malformations in 46% of fetuses whereas in the rest only hindlimb oligodactyly was present [5], PBN pretreatment was less efficient despite the same dosage and timing of 5zaC application and we may suppose that the PBN concentration was suboptimal.

### Global DNA methylation

We have been able to confirm the DNA hypomethylating effect of 5azaC in embryonic limb buds by pyrosequencing a widely spread global DNA methylation marker from the rat noncoding DNA [short interspersed nuclear element (SINE), rat ID element] [47]. To put that positive result in a proper context, it must be stressed that by platforms such as Illumina 450K, which were developed only for the human genome, the effects of 5azaC could not be associated with the DNA methylation levels of activated ERVs from the noncoding DNA, because the platform does not contain those annotations [39]. Treatment with 5azaC induced a statistically significant hypomethylation and pretreatment with PBN significantly normalized global DNA methylation only in developmentally less advanced embryonic hindlimb buds [42], although the improvement of malformations in fetuses was similar in both types of limbs. The reason for such improvement may be the known robustness to perturbations of the network structure that regulates early limb development [55]. Sometimes, 5azaC did not change DNA methylation level, although it changed developmental parameters such as proliferation [61]. Indeed, a recent review discusses many reasons why DNA methylation is still not a marker predictive of response to DNA hypomethylating therapy [62].

### Cell proliferation

We now found that DNA hypomethylating agent 5azaC applied twice during pregnancy was able to diminish cell proliferation in limb buds of embryos of gestating dams treated in vivo, which confirmed our previous result on limb buds cultivated in vitro with 5azaC [63] or its activity discovered in cancer cells [64].

In contrast to the negative 5azaC activity on cell proliferation in the limb bud as an immature whole organ, we showed that proliferation of the vertebral cartilage was increased by 5azaC, similarly as in 17-day-old fetal epiglottis of the same strain of rats transplanted in vivo [65]. The 5azaC-induced hypomethylation also reversed the aged phenotype of the adult mesenchymal stem cells by enhancing cell proliferation [59].

These differences in the impact of 5azaC in various types of cells suggest that the effect of changes in DNA methylation on cell proliferation is organ and cell-type specific. In accordance to that is the recent genome-wide DNA methylation analysis by whole genome bisulfite sequencing that demonstrated no global changes or large-scale hypomethylated blocks in cell cycle phases of the human early passage primary dermal fibroblasts, although authors discuss that the situation might be different in more proliferative cells such as stem cells or cancer cell lines [66].

Importantly, although PBN pretreatment of 5azaC-treated dams neither improved proliferation in embryonic limb buds nor diminished apoptosis in the vertebral cartilage or extremely elevated apoptosis in the liver, it normalized proliferation in the liver that was significantly diminished by 5azaC. Although patients with MDS and hepatic impairment were excluded from the clinical trials on Vidaza [67,68], the FDA-approval summary on the MDS treatment with 5azaC (Vidaza) mentioned “liver function abnormalities in 16% of patients with hepatobiliary disorders and in two patients with previously diagnosed liver cirrhosis” [67,68]. Therefore, the possible beneficial activity of PBN pretreatment seems to be worth further investigation, and more so because it was found that 85% of MDS patients expressed high levels of ROS [69].

## Conclusion

We discovered that the spin-trap free radical scavenger PBN significantly ameliorates the pronounced negative impact of a DNA hypomethylating drug 5azaC on the normal development of offspring during the mammalian gestation. Therefore, the mechanism of teratogenic activity of the 5azaC partially depends on activation of ROS/RNS. As PBN neither diminished apoptosis nor enhanced cell proliferation of immature tissue under the influence of 5azaC, its therapeutic impact on elevated free radical levels in MDS and related diseases may be of interest. That PBN restored proliferation in the liver may also be of interest for the treatment of hepatotoxicity and liver regeneration/transplantation strategies [44].
